# Professional Identity Formation Metaphors: Old Problems and New Promises

**DOI:** 10.5334/pme.1803

**Published:** 2025-05-05

**Authors:** Lara Varpio, Marije van Braak, Anne de la Croix, Adam P. Sawatsky

**Affiliations:** 1 Department of Pediatrics, Perelman School of Medicine at the University of Pennsylvania, US; 2 The Children’s Hospital of Philadelphia, US; 3 Department of Language, Literature and Communication, Utrecht University, Utrecht, the Netherlands; 4 The Team Research in Education, Amsterdam UMC, Faculty of Medicine, Vrije Universiteit Amsterdam, The Netherlands; 5 Division of General Internal Medicine, Mayo Clinic, Rochester, MN, USA

## Abstract

**Introduction:**

While medical education scholars have made important contributions to our understanding of professional identity formation (PIF), some ways of thinking stubbornly endure despite being repudiated in the literature. We argue that two metaphors embedded in the PIF discourse contribute to obstructing change: PIF IS A JOURNEY and PIF INVOLVES FITTING INTO A MOLD.

**PIF IS A JOURNEY:**

This metaphor assumes that each learner’s PIF work starts the day they enter medical school; that their pre-matriculation PIF work isn’t part of their PIF journey; and that the paths to PIF are equally accessible for all. We argue that this is not the case. Drawing on Appadurai’s theories of aspiration, we critique this metaphor for neglecting each medical learner’s unique cultural backgrounds and aspiration journeys.

**PIF INVOLVES FITTING INTO A MOLD:**

This metaphor perpetuates expectations that medical learners and practicing physicians ought to adopt medicine’s norms. It places the blame for the crises and dissonance individuals experience when adapting to medicine’s culture on the individual—their failure to fit in. While standardization is necessary for maintaining professional standards, it should not suppress individuality, limit diversity, nor constrain adaptability.

**PIF AS FUNCTIONAL CRAFTING:**

We propose that this new metaphor balances standardization with individuality in PIF. It makes room for both the enjoyable and challenging aspects of PIF, and allows for unique, personalized professional identities within the medical field. We propose that this new metaphor aligns with subjectification, an orientation that can encourage flexibility and respect diversity in professional identities.

## Introduction

‘*Metaphors are fine if they aid understanding*,

*but sometimes they get in the way*.’

-Richard Dawkins

Medical education’s research literature is rife with manuscripts wherein professional identity formation (PIF) is summarized [1], theorized [2], problematized [3] and revolutionized [4]. In this body of work, scholars have thoughtfully considered professional identity, its formation, and processes for nurturing individuals to think, act, and feel like a physician [5,6]. And yet some ways of thinking tenaciously endure despite being actively refuted in the literature. While we recognize the inevitable lag between the time new knowledge is disseminated and the uptake of that knowledge in practice, we propose that there is another barrier preventing change: our metaphors. These metaphors—embedded within the discourse about professional identity and its formation—shape our conceptualizations and our understanding. They facilitate some ways of thinking about PIF, while simultaneously impeding others. We contend that these metaphors risk hampering learners’ progress at every stage of the educational continuum. By allowing them to persist uncritiqued, we risk doing harm to those we seek to nurture into our professions.

The need to consider how medical education shapes the professional identities of physicians has been discussed in scholarship dating as far back as 1957 [5]. Sociologists highlighted the requirement for each physician to develop a professional identity [5,7] underpinned by specific conceptualizations of expertise, quality, and service [8]. The medical professionalism movement recognized that a strong sense of professional identity was important for dealing with the complexity and ambiguity of a physician’s work [9]. Subsequently, PIF was established as a pillar of medical education [10]. While experts identified the individual, interactional, institutional, and discursive nature of identities [11,12], much of medical education’s PIF literature focused on the individual and developmental nature of identities, focusing on psychological framings [13,14,15]. Within the last decade, a broadened view of identities—including interactional [16], sociocultural [17,18], discursive [19], and critical [20] approaches—has begun to challenge older assumptions about and conceptualizations of PIF.

Across this history, the discourse is rife with metaphors that have helped us to wrestle with the abstract and intangible nature of PIF, to think about PIF theoretically, and to realize it in practice. These metaphors are powerful. They are not merely forms of expression. Modern literary theories argue that “metaphor is the main mechanism through which we comprehend abstract concepts and perform abstract reasoning” [21, p.244] Therefore, to understand abstract phenomena (like PIF), we rely on metaphors that invoke large networks of relevant knowledge [21] they both solicit ways of thinking (and so help us to think about PIF) but also restrict our ways of thinking (and so limit the diversity of ways we can think about PIF) [22]. In other words, today’s language theories advise us to attend to the metaphors that shape the ways in which we think about PIF [23,24,25] because: (a) the metaphors that shape our thinking may be inadequate, but (b) we are unaware of the metaphors’ shortcomings and their contribution to limiting effects on research since metaphors largely go unnoticed [22]. The continuous conventional use of metaphors “has deadened our ears” [23, p.245] to the fact that they are untested. And so, we argue that it is essential to critique the PIF metaphors that often evade analysis because each metaphor encourages one way of thinking about PIF but also, simultaneously, inhibits other ways of thinking about PIF. As Burke warned [26], every way of seeing a phenomenon emphasizes one way of thinking but also obscures other ways of thinking.

As four scholars engaged in different PIF-related programs of research, we have been frustrated with two metaphors that have promoted some ways of thinking about PIF, and that are proving particularly hard to overturn despite growing bodies of evidence refuting them. Therefore, based on our years of research (L.V., M.v.B, A.d.l.C., A.S.) and on clinician educator experiences (A.S.), we highlight two tenacious metaphors that are particularly important to critique: PIF IS A JOURNEY and PIF INVOLVES FITTING INTO A MOLD. First, PIF has been framed as a journey that begins the day a learner matriculates into a medical training program. We contend that this metaphor discounts the PIF work learners undertake before they begin medical school. For many learners, PIF is not a charted journey along a clear path; instead, PIF begins well before acceptance letters are received and requires clearing their own paths through largely unknown terrain. Second, professional identities have been conceptualized as molds into which individual learners should fit. In alignment with others, we argue that PIF ought not to be thought of as a rigid structure our learners must fit within. Instead, it is more usefully conceived of as a malleable construction that can encourage and respect variations across individuals’ professional identities. Finally, we end this paper by presenting a new metaphor that might help educators eschew these problematic ways of thinking: PIF AS FUNCTIONAL CRAFTING. We propose that the concept *subjectification* can help us realize this crafting work.

We acknowledge that this new metaphor also imposes limitations. Therefore, we offer this manuscript as a reflection on our field’s ongoing study of PIF: while old ways of thinking need to be modified to make room for new insights, our new metaphors are similarly not problem-free.

## PIF IS A JOURNEY

‘*Welcome to medical school!*

*Today begins your professional identity development journey towards becoming a physician*.’

Words of welcome like these are often delivered to greet incoming classes of medical students. But embedded therein is a metaphor: PIF IS A JOURNEY. This metaphor carries assumptions: that each learner’s PIF work starts the day they enter medical school; that the work they did before matriculation isn’t part of their PIF journey; and that the paths to PIF are equally accessible for all. We argue that this is not the case. Medical education scholars have frequently studied PIF at the undergraduate [27,28] (UME), graduate [29] (GME), continuing medical education [30] (CME) levels, and across these levels [31,32]. Less frequently have we acknowledged and appreciated how an individual learner’s PIF journey begins well before matriculation. Faihs et al noted that PIF “is a life-long process, starting even before professional education” [33]. However, despite this recognition, the medical education literature has tended to examine professional identity and its formation as it occurs within the walls of our medical schools and academic teaching hospitals. We expect that this focus is rooted in practical considerations—it is straightforward to solicit research participation from learners and faculty members within these institutions; it is much harder to identify, communicate with, and secure participation from people outside these walls. While expedient, this approach contributes to perpetuating the PIF IS A JOURNEY metaphor and the problematic assumptions embedded therein.

Medical education has long acknowledged that each matriculating medical student is a unique, contextually bound individual who comes to the profession from their own history, culture, and pre-existing identities [14]. Therefore, each matriculant’s PIF journey is understood to be unique. Each learner constructs their physician identity in interaction with learners and faculty who are also on their own unique professional identity journeys. However, obscured by the PIF IS A JOURNEY metaphor is the fact that PIF work is tied to the culture that each individual is born into and to the aspirations embedded therein. Physicians’ PIF experiences start well before they even apply to medical school. When we ignore their pre-matriculation travails, we risk diminishing the staggering effort some individuals exert before beginning medical training.

One example of how pre-matriculation experiences interact with medical training’s PIF work is related to cultural aspects of being. Appadurai [34] proposes that the cultures an individual is born into and lives in significantly impact the individual’s capacity to aspire to a given career. Appadurai notes that academics have tended to frame culture historically—i.e., as the unique system of norms, values, beliefs, and traditions that form the foundation of specific communities. From this perspective, culture is a heritage that each individual carries within them; it is a person’s history through which they live today and through which they conceive of the future. Appadurai cautions that we have neglected to appreciate how aspirations are part of *cultural* norms [34]. The aspirations that an individual has for the future are shaped by the culture(s) within which the individual lives, is educated, and is recognized; aspirations are part of the map of culturally generated ideas and beliefs that influences the horizon of things an individual aspires to achieve. While individuals can aspire beyond their cultural norms, Appadurai’s theory proposes that *my* aspirations are informed by what *we* see as possible.

Appadurai explains that, since it is culturally shaped, the capacity to aspire is not evenly distributed across society, nor across individuals. An individual’s aspirations are shaped by *all* the cultures (e.g. national, socio-economic, gender) to which they belong. Aspiring to something outside of one’s cultural groups requires creating new justifications, new narratives, and new pathways that are not part of the individual’s cultural context.

Applying Appadurai’s theory to the aspiration of becoming a physician highlights how each medical learner begins their PIF journey *well before* matriculation. For some, that navigation was straightforward. Their cultural backgrounds (often scaffolded by the experiences of physician-trained family members) already included narratives of how to get into medical school, stories of what the journey would be like, and pathways for getting there. For these learners, navigating the aspiration to be in the profession was a well-trod path with many nodes and malleable pathways. However, for other learners, the map of nodes and pathways to becoming a physician was not charted. For these learners, the horizon of possibilities was more brittle and more tenuous, with fewer nodes and more rigid pathways [34]. Appadurai argues that this difference is *not* because those with less privilege are in any way personally or culturally deficient. Instead, he argues that different cultures have generated different maps that are part of the cultural history within which individuals live and generate future aspirations. Therefore, if an individual aspires to something (i.e., being a physician) that isn’t part of their cultural map, realizing this aspiration requires *more work*. For these learners, just getting to the point of matriculation involved migrating across cultures with maps that didn’t have all the routes, nodes, and pathways inked therein.

When we uphold and act through the PIF IS A JOURNEY metaphor, we ignore (and perhaps even negate) the work that some learners undertake just to arrive at the doors of our medical schools. If each medical learner is unique, then we must recognize that they are not all starting their PIF journey at the same starting point and the paths available to them are highly variable. Some matriculants have already traversed enormous cultural distances. By ignoring these differences, we fail to appreciate how some learners arrive at matriculation already travel weary.

## PIF INVOLVES FITTING INTO A MOLD

‘*I don’t like the person I’m becoming*.’

In studies of UME and GME learners’ PIF experiences, concerns like the one above have often been anonymously shared. Part of a trainee’s PIF work involves adapting to the medical culture. This culture has norms, values, and beliefs that may not align with—and may even contradict—the cultural maps learners held upon entering their professional education. And yet, while research is increasingly reporting the harms some learners experience during their PIF work, there remains an old, persistent expectation that learners ought to adopt medicine’s PIF norms. We suggest that this way of thinking is rooted in a metaphor that is often not explicitly stated: PIF INVOLVES FITTING INTO A MOLD.

Most physicians-in-training and physicians-in-practice aspire to be labelled as a “good doctor”, but too often we fail to consider the many assumptions embedded therein. And so “the good doctor” identity risks becoming an inflexible and unchanging mold, and, through PIF work, medical learners contort themselves to fit into this mold. We argue that this metaphor must be critiqued and new practices adopted. We are *not* advocating for abandoning all expectations of standardization in medical education; indeed, to retain the responsibility, accountability, and status as a self-regulating profession there needs to be standards, expectations, and shared ideologies. Instead, we argue that the ways in which we accept and enact ideas of professional conformity is problematic. Such expectations for standardization risk assuming that PIF work involves fitting into the profession’s professional identity mold. But such expectations are problematic.

This idea of a professional identity mold might stem from what Frost and Regher called a discourse of standardization that they connect to competency-based education “which explicitly seeks to educate students towards specific standards as they are articulated in core competencies” [35]. This discourse of standardization can drive even the most complex, invisible, personal, and context-dependent concepts into measurable tick-box exercises with clear desired outcomes [36,37]. The move towards standards, clear outcomes, and measurability has many appealing qualities—e.g., demonstrating the attainment of competency benchmarks and fulfilling the contract with society. And yet, this focus on standardization does not always yield outcomes we hope for in practice [37] nor in education [37,38]. As Frost and Regher noted, and as ten Cate et al. echoed [39], standardization must be balanced with respect and preservation of diversity, and with the valuing of professionals as unique individuals.

To be sure, we must uphold standards, procedures, and knowledge in the medical profession. However, we suggest that these standards, procedures, and knowledge must continuously be questioned and critiqued; they cannot become the assumed “norm”. The literature suggests that learners do not always feel empowered to engage in such questioning and critique. Research shows that medical learners work hard to meet expectations so that they can join the profession; they behave in ways that are “socially desirable: being professional at the right time, the right place, towards the right people” [40]. This strategic behavior illustrates students’ awareness of the mold they are expected to fit into, and their efforts to manage their self-image in accordance. This identity work is even more laborious for minority students because the mold was not crafted based on their experiences and values; it was constructed over time by those who were dominantly present in the profession [3]. When we allow the PIF INVOLVES FITTING INTO A MOLD metaphor to persist, we create a situation where standardization is demanded, where students perform conformity, and where there is little room for diversity.

When we uphold this metaphor, real problems are generated. First, when the mold is inflexible, we fail to allow for a range of expressions of the physician identity. If we truly want a more diverse population represented in the medical profession, we need to make room for individuals to find ways to both meet standards of expectations *and* to honor their unique identities. Second, when the mold is inflexible, it doesn’t allow for adaptation to the changing landscape of medical practice. Just as the COVID-19 pandemic demanded adaptive expertise, future crises will do the same [41] and demand skills that we have yet to recognize. The uncertainties of future practice require that we make room for adaptability [42] in physicians’ professional identity.

We acknowledge that our community is increasingly aware of the PIF mold and is working to make it more malleable. Indeed, change is happening. But metaphors are powerful because they are pervasive but inconspicuous. When the PIF INVOLVES FITTING INTO A MOLD metaphor remains in our educational practices, we risk leaving our learners to think that something is wrong with *them* if they don’t fit within the profession’s expectations. This is misleading at best, and irresponsible at worst. While research highlights the need for flexible expressions of professional identity [35,43,44,45,46], the hidden assumptions embedded in the metaphors we uphold in practice often recreate expectations of an unchanging mold. Fortunately, there is a growing chorus of scholars arguing for a change to the ways in which we describe, define, and use the concept of professional identity and the work of its development. Sternszus et al. implore educators to critically re-examine professional culture and norms that have contributed to these difficulties [44]. We are starting to see changes in individual institutional practices, but broader scoped efforts are needed. Such efforts would require physicians to band together to do the *collective* work of reimagining medicine’s professional culture and identities [47] and to implement that new imagining. This reimagining would need to be far-ranging. For instance, some have argued for socializing physician trainees to function as professionals who can disrupt broken systems and allow physicians to fulfill their mission to patients [48,49]. Others, looking at the problem from an interpersonal level, have called for recovering the importance of relationships in medical education, protecting them from market forces that prioritize their labor and constrain the development of human connection [16,50,51]. But, while we work to change the culture of medicine, we must also change the metaphors shaping our practices.

## A new metaphor: PIF AS FUNCTIONAL CRAFTING

‘*I need to bring my whole self to work. It makes me a better physician. My professional identity is continuously and dynamically evolving through all my professional interactions*.’

As we work to see past the constraints of old metaphors, new metaphors are needed to highlight ways of thinking that were previously obscured. However, we acknowledge that any new metaphor will, at best, be a temporary solution—one that will encourage a new way of thinking while simultaneously hiding other ways of thinking. Therefore, we offer a new PIF metaphor that may temporarily offer new insights and affordances: PIF AS FUNCTIONAL CRAFTING. Functional crafting can be defined as the work of making creations that are both functional and aesthetically pleasing [52]. It is a broadly inclusive term, encompassing crafts as diverse as wood working and furniture building, to knitting and sewing, to throwing clay pots and dishes [53]. Functional crafting involves an individual or team making a creation that is designed to serve a purpose with an aesthetic in mind [53]. We find two elements of this metaphor particularly useful for highlighting aspects of PIF that have been constrained by other metaphors.

First, PIF AS FUNCTIONAL CRAFTING supports the need for PIF to both be constrained by standardization *and* be open to flexibility. Crafts are often sold as kits where, if you carefully follow the instructions provided, the creator can turn a set of pre-structured pieces into a finished work (e.g., the crafter follows the instructions to turn pre-cut pieces of wood into a birdhouse). However, crafting can also take place without kits, where the crafter transforms raw materials to a finished work (e.g., the crafter wields a whittling knife to turn a tree branch into a birdhouse). In these ways, functional crafting is regulated—be it by the kit instructions, by the function the object must fulfill, or by the resources available—and also creative. The PIF AS FUNCTIONAL CRAFTING metaphor frames PIF as an act of subjective creativity within the constraint of professional standards. In this metaphor, PIF work involves providing individuals with the materials of the physician identity, explaining the final functional structure to be erected (i.e., professional competencies, mandates, and expectations), and then allowing them to uniquely craft their own structure—with the materials the profession provided *and* with the individual’s personal stock of materials, with some materials being left on the crafting table and unique materials being identified and included. In this metaphor, the work of the educator is to help the learner craft their unique creation so that it meets the standards of the profession in ways that are as idiosyncratic as the creative visions of the learner. The responsibility of the profession is to allow each physician to simultaneously uphold *and* push the boundaries of standard expectations—to question the materials provided, to challenge the functional expectations, and to make it possible for each physician’s unique creation to push the boundaries of what “should” be part of the physician’s professional identity.

Second, the PIF AS FUNCTIONAL CRAFTING metaphor makes room for PIF work to also be enjoyable. Recent research has highlighted how PIF is laborious. Creating one’s own professional identity—of melding unique subjective aspects of one’s identity with the structures and expectations of the profession—requires work [54,55]. The process will be transformative—it will shape who individuals become as people and professionals—but for many medical trainees the PIF work will not be harmless [43,56,57,58,59]. As this body of research illustrates, forming a professional identity can be hard work, fraught with unpleasant, even threatening experiences. This fact is not negated when we also acknowledge that PIF experiences can also be joyous. Thousands are called to join the medical profession, and so it stands to reason that PIF experiences can be pleasing. Therefore, we need a metaphor that recognizes the personally difficult work of PIF *as well as* the pleasure that can come from PIF work. A physician’s career is full of PIF experiences. Any one of these might be damaging, innocuous, or joyous. We need a metaphor that makes room for PIF experiences to land anywhere across this continuum. We suggest that the PIF AS FUNCTIONAL CRAFTING metaphor offers such a way of thinking.

## Realizing PIF AS FUNCTIONAL CRAFTING Through Subjectification

We suggest that realizing the PIF AS FUNCTIONAL CRAFTING metaphor requires affording greater importance to the subjectivity of each individual medical learner and practicing physician. This approach would focus on what it means to *exist as* a physician, and on the way each person shows up as *subject* in specific contexts. This approach draws attention to *subjectification*.

Subjectification was originally introduced by Biesta as one of the three goal domains of all education [60]. One goal is to aim for *qualification*: to provide students with “knowledge, skills, and understanding that will qualify them to do something” [49]—i.e., to enable learners to gain the knowledge, skills and attitudes required to meet competency requirements. A second goal is to *socialize* students into the profession, into its norms, values, practices, cultural aspects, and traditions [49]. Current thinking about PIF is largely aligned with this second educational aim [61]. Qualification and socialization are essential, but they are not sufficient. We miss something when students graduate being just qualified and socialized medical professionals. We need to work with and through standards (qualifications) and norms, values, and practices (socialization), but we also need to work beyond them to educate individuals with agency who shape their qualifications and socialization into something more. This is precisely what subjectification is all about.

Subjectification, as the third educational aim, refers to people becoming visible as subjects [60] in situations in which *who they are* is at stake, in which *they uniquely are being hailed*, in which it matters that *they*—specifically them and no one else—are there. Such situations are often grey areas [62]: situations in which several directions can be taken. In such situations, *the person being hailed* has the freedom and responsibility (a) to choose *a* direction for action [63], (b) in response to the specificities of *a* situation, and (c) from *their* subject position. By definition, then, such a response is unique in that it is irreplaceable—it can neither be replicated nor can it be recreated. Responding to the situation provides evidence to *how one unique physician acts*; it shows how, in *this* situation, *this* physician took *this* action with their unique identity. In other words, this particular response, in this unique situation, at this particular moment, shows why it matters that *I am here* and that *I am called to act* [61,62]. Put differently still, subjectification adopts a fuller perspective on identity formation: it is *what I do* with *who I am* in *relation to others* in *this situation*. It is important to note that this is not a shift back to professionalism’s focus on behaviors and rules of conduct; instead, it is about a more flexible, unique, and creative conceptualization of identity in action, constructed in the moment.

For medical learners, becoming visible as subjects means acting in a medical situation from one’s unique subject position [62]. Acting as a unique subject requires medical learners to work through their “singularity, irreplaceability and particularity” in concert with others in the situation [64]. This requires the learner to identify with and become part of the medical profession, *and* to redefine that identity as a *unique self within the profession*. It involves embracing their subjectivity in the context of professional normativity. It involves agentic action in the constraints of standards [49]. The medical profession has norms, standards, and ways of being and doing that are upheld, replicated, and enacted by its members. Positioning oneself in a particular configuration of those norms, standards, and ways of being and doing requires nimble navigation [65]. It requires deep familiarity with professional traditions and expectations so that the individual can take calculated actions of responsible freedom [66].

For example, scholars have asserted that “the maintenance of appropriate boundaries in the doctor-patient relationship has been recognized as a core element of medical professionalism [67 p. 547]”. And so, if a resident discloses having invited a lonely refugee patient to a family holiday dinner, the educator has many reactions to consider. One might critique the resident’s decision as unprofessional thereby reinforcing the professional norm. One might discuss the resident’s invitation as an act of generosity. One might explore with the resident how this event is an enactment of their unique subjectivity [68] within the norms of the medical profession. One might bring all these reactions (and others) into the discussion. Education towards subjectification would create a space where the sharing of this unusual (and, arguably, *risky* in terms of professional behavior) event is observed and analyzed as an interruption of the status quo *and* as an agentic act of a subject who is finding room to be their unique self within the profession.

Subjectification draws our attention to the friction [61,69] that exists between what the medical profession expects and what the individual physician does. Awareness of this friction can create a space where learners see and discuss the discrepancies between these two positions. It creates a space where identity struggles, ruptures, and navigation can be appreciated as a normal and valuable part of PIF. It creates a space within which the individual can craft a balance between standardization and their unique identity [70]. Subjectification affords new justifications, new narratives, and new pathways. It opens possibilities for (re)negotiating the status quo [71]. In this orientation, learners are positioned as subjects co-creating their education, not objects passively accepting medical education’s lessons. This requires educators to make room for “subject-ness as something that can emerge” [68 p. 761]. It can emerge during any topic of discussion or patient engagement. For example, in classes addressing doctor-patient communication, the educator might encourage discussion about “diverging” from standardized scripts and protocols—not to celebrate an *anything goes* casual communication approach, but instead to empower students to be responsibly creative, to engage in functional crafting.

## Conclusion

We have identified two metaphors that, despite being critiqued, have persisted and remain as part of our medical education discourse: PIF IS A JOURNEY and PIF INVOLVES FITTING INTO A MOLD. We suggest that these metaphors can do real harm to physicians-in-training and to physicians-in-practice. We offer a new metaphor—PIF AS FUNCTIONAL CRAFTING—that can offer up new ways of thinking about PIF. This new orientation will require medical educators, together with their learners, to face old PIF traditions, to learn to craft standards and diversity together in unique ways that, while arduous, can also be joyous. We propose that *subjectification* can be a helpful goal domain that helps to realize this crafting work. The difficult work for medical educators is, therefore, no less than to supervise students’ negotiations with the physician identity and to promote an outcome that preserves both the profession and the learner [62], and to respect the dangerous work *and* the joy that can be part of PIF. Truly no small feat. We acknowledge that the PIF AS FUNCTIONAL CRAFTING metaphor imposes restrictions on our thinking just as previous metaphors did. We look forward to discussing the affordances and weaknesses of this metaphor with the medical education community of PIF-interested scholars. But regardless of the strengths and weaknesses of the metaphors we craft, we argue that creating space for the self *and* the profession, for the personally difficult work *and* the joy of the work is essential for offering new levers for creating medical professionals who are agentic subjects of their own professional actions [49].
